# Quality of life and functional outcome in Swedish children with low anorectal malformations: a follow-up study

**DOI:** 10.1007/s00383-018-04431-8

**Published:** 2019-02-07

**Authors:** Helena Wigander, Margret Nisell, Björn Frenckner, Tomas Wester, Ulf Brodin, Maria Öjmyr-Joelsson

**Affiliations:** 1 Department of Women’s and Children’s Health, Karolinska Institutet, 171 76 Stockholm, Sweden; 2 Division of Pediatric Surgery, Astrid Lindgren Children’s Hospital, Karolinska University Hospital, 171 76 Stockholm, Sweden; 3 Department of Health Sciences, The Swedish Red Cross University College, Huddinge, Sweden; 4 Division of Child- and Adolescent Psychiatry, Department of Women’s and Children’s Health, Karolinska Institutet, Stockholm, Sweden; 5 The Medical Statistic Unit, Department for Learning, Informatics, Management and Ethics, Karolinska Institutet, Stockholm, Sweden

**Keywords:** Low anorectal malformations, ARM, Quality of life, QoL, HAQL, BFS

## Abstract

**Purpose:**

The aim was to investigate the quality of life and bowel function in children with low anorectal malformations (ARM).

**Additional aim:**

To evaluate the Swedish version the Hirschsprung’s Disease/Anorectal Malformation Quality of life Questionnaire (HAQL).

**Methods:**

Forty-four children and their parents were invited to complete the HAQL and the Bowel Function Score (BFS). Healthy children participated as controls and completed the HAQL.

**Results:**

Seventeen children and 18 mothers completed the HAQL. The children reported impaired function in the physical symptom (PH) fecal continence (FC) and laxative diet (LD) domains compared to controls. Compared with their mothers, they reported impaired physical function and more symptoms in the emotional functioning (EMF) and PH domains. 27 families completed the BFS; 63% reported normal bowel function, 33% moderate outcome and one patient, comprising 4%, poor outcome. Evaluation of the HAQL, FC, EMF and PH domains showed no obvious conflicts.

**Conclusions:**

The children did not differ much regarding their QoL, even though they appeared to have impaired bowel function and worse emotional functioning compared to controls. The mothers underestimated their children’s physical symptoms and overestimated their emotional functioning. Evaluated domains in the HAQL appear to work as intended, but the questionnaire needs further development.

## Introduction

Anorectal malformations (ARM) are congenital anomalies involving the anus and the rectum and varies from minor to more complex with an incidence in Sweden of 1:3000 [1]. Despite corrective surgery during infancy, the children often experience varying degrees of functional problems such as constipation or incontinence [2]. A traditionally long-term outcome of low anorectal malformations has been considered good in most of the patients [3]. However, it has been shown that it is common for patients with low ARM to also experience constipation as adults [4], although the bowel function often improves over time. An effective treatment of constipation is essential to optimize bowel function [5].

Studies concerning bowel function and quality of life (QoL) in children with low ARM are limited, although it has been shown that bowel function problems may significantly impact a person’s QoL, and the degree of severity of fecal incontinence affects QoL [6]. Adolescents with ARM were found to have considerable intestinal symptoms, which also influenced their QoL [7]. Furthermore, adult patients with ARM reported impaired QoL compared to healthy peers [8].

The concept of QoL concerns an individual’s satisfaction in all aspects of life and is a broad holistic concept constituting a multidimensional assessment of a person’s current life [9, 10]. When assessing a person’s health status, focusing on aspects of health care, the term health-related quality of life (HRQoL) is used [11].

Generic QoL questionnaires are available in Swedish, but disease-specific HRQoL evaluation tools provide us with a better sensitivity and specificity as they focus on specific problems and reveal the effects of the distinctive symptoms of a specific disease [11]. The disease-specific tool HAQL (Hirschsprung’s Disease Anorectal Malformation Quality of life Questionnaire) was developed in the Netherlands [12] and has been translated and culturally adapted into Swedish [13]. The Swedish version of the HAQL is the only disease-specific questionnaire available for this category of patients in Sweden. The HAQL has also been translated and used in Italy and France [14, 15]. The HAQL was developed for both ARM’s and Hirschsprung’s disease, but the current study is focused on children and adolescents with ARM. The Swedish version of HAQL has not previously been used and tested among children with low ARM.

The purpose of the current study was to investigate the quality of life and bowel function in children with low ARM. An additional aim was to evaluate the Swedish version of the disease-specific questionnaire for children and adolescents (HAQL).

## Methods

### Participants

**Fig. 1:**
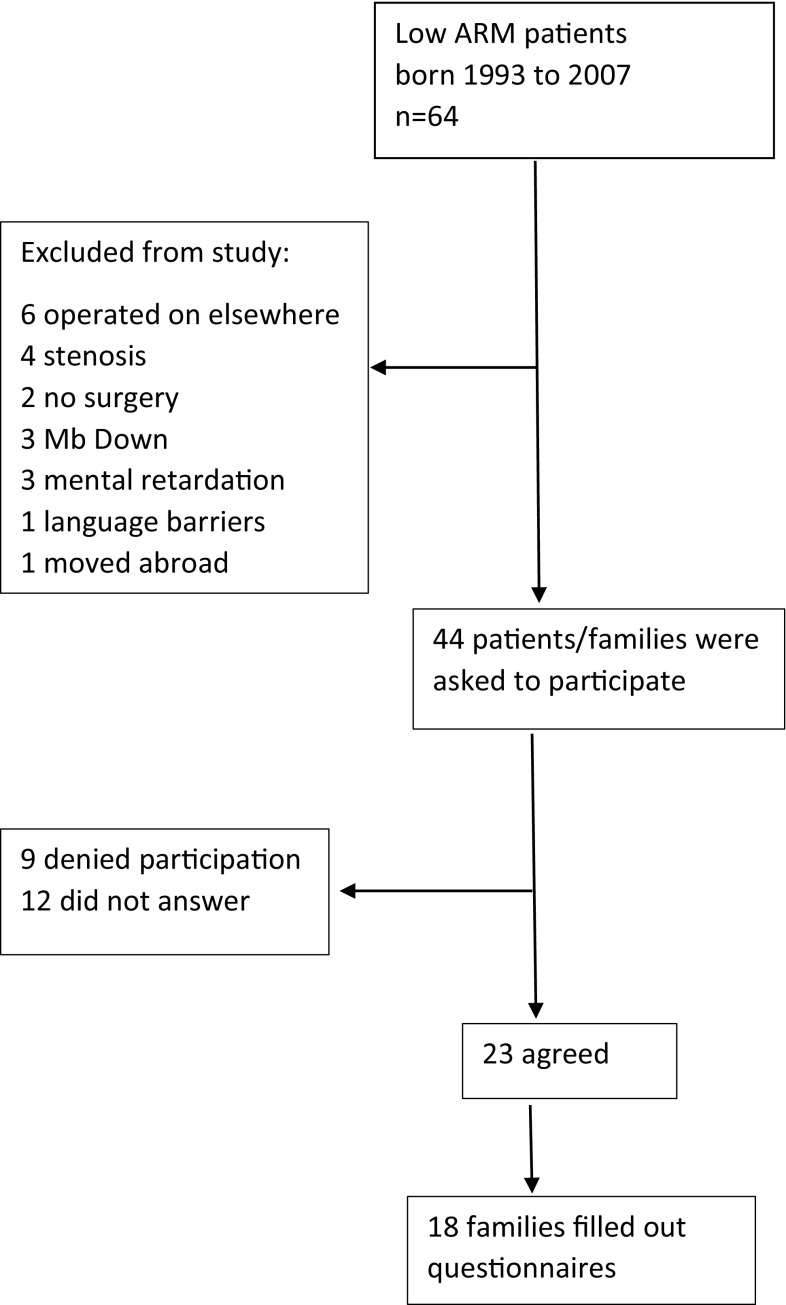
Flowchart of participants

The index group comprised all children with low ARM born between 1993 and 2007 who had been cared for at Astrid Lindgren Children’s Hospital, Stockholm, Sweden. 64 patients were identified through hospital records. 20 of these patients were excluded. 44 families were contacted via postal mail; please see Fig. 1. Reminders were sent out twice. The timeframe for data-collection of the HAQL was ongoing from 2011 to 2015 and patients were included after they had turned 8 years of age. A control group comprising healthy children who had visited Astrid Lindgren Children’s hospital for a minor procedure was used for comparison. In addition, in 2015, the patients in the index group described above were asked to complete the Bowel Function Score (BFS).

### The HAQL questionnaire

Quality of life was assessed using the Swedish disease-specific quality of life questionnaire, HAQL (Hirschsprung’s Disease and Anorectal Malformation Quality of life Questionnaire). There are three age-specific versions of the questionnaire (8–11, 12–16 and 17 + years) and proxy versions for the two younger age groups. HAQL comprises 39–42 items including 11 domains with questions concerning laxative diet (LD) (2 items), constipating diet (CD) (2 items), presence of diarrhea (PD) (2 items), constipation (CON) (1 item), fecal continence (FC) (8 items), urinary continence (UC) (4 items), physical symptoms (PH) (9 items), body image (BOI) (2 items), social functioning (SOF) (3–5 items), emotional functioning (EMF) (6–7 items) and questions concerning sexuality for the older age-groups (SEF) (2 items). Each item consists of two parts: (1) the respondent indicates how often a specific problem occurs over a 1-week timeframe, scored on a four-point scale ranging from (never, sometimes, often, very often). (2) How unpleasant it was for the respondent, scored on a four-point scale (not at all, a little bit, unpleasant, very unpleasant). HAQL is a disease-specific QoL questionnaire developed in the Netherlands for children and adolescents with fecal incontinence [12]. The HAQL is also available in Italian, English and a French version [14–16]. The HAQL has been translated and culturally adapted for Swedish settings [13].

### Bowel Function Score

Bowel function was assessed using a seven-item Bowel Function Score (BFS) [17, 18]. The BFS is a scoring method that assesses voluntary control of defecation sensation, frequency, soiling, constipation, and social impact on the individual. Parents completed the score for children less than 16 years of age. A total score of 17–20 is considered normal bowel function in a normal range population [5, 17]. The BFS has been validated for patients with ARM [19, 20].

### Statistical methods

For the evaluation of the HAQL questionnaire, a Mokken scalability analysis [21] was applied to evaluate the possibility of the items’ creating a sum score for each domain in the questionnaire and to verify that the item scalability, *H*_*i*_, was positive for item ‘*i*’ within the domain. The FC, EMF and PH domains comprise 8, 6 and 9 items, which allow the domains to be subjected to a basic evaluation that considers the feasibility of a sum score approach. A supplementary parsimonious Rasch analysis [22] was carried out for further evaluation of the sum score approach. For the FC, EMF and PH domains, 34 questionnaires (17 children with ARM and 17 mothers’ questionnaires) were transformed into Rasch measures. For comparison between the child and the mother, the Wilcoxon matched pairs test or a paired *t* test were used. For comparison of patients and the control group, the Mann–Whitney test for independent groups was used. In some domains where the data predominantly included only low or zero scores, the Fisher exact test was used. A Mokken scalability analysis and a Rasch approach were applied for BFS. The agreement with HAQL was estimated using the non-parametric Spearman rank correlation based on 16 pairs. *p* < 0.05 was considered statistically significant. Mean and standard deviation is presented as mean (SD).

### Drop-out analysis

A drop-out analysis was conducted using hospital records and data on bowel function from the latest follow-up. The t test was used for comparison between participating children with ARM and non-participating children with ARM.

## Results

### Patient characteristics

23 (52%) families agreed to participate, 17 children, 18 mothers and 13 fathers returned consent forms and completed HAQL questionnaires; please see Fig. 1. A control group comprising 17 children, 16 mothers and 7 fathers of healthy children also completed the HAQL questionnaire. 27 families (61%) returned completed BFS questionnaires. 16 families completed both the HAQL questionnaire and the BFS questionnaire. For further characteristics see Table 1.

**Table 1:** Demographic data for index group and control group treated at Astrid Lindgren Children’s Hospital

	All patients in index group	All control patients	Participating patients completed HAQL (child + mother)	Participating control patients completed HAQL (child + mother)	Participating patients completed BFS	Non-responding patients (denied participation or did not answer)	Non-responding control patients (denied participation or did not answer)
Number of patients	44	50	17*	17	27	16	33
Sex							
Male	22	35	8	11	15	8	24
Female	22	15	9	6	12	8	9
Age							
Mean		11.7	11.2	11.7	14	11.2	11.6
Range		7.6–17.9	7.4–18.3	7.5–17.9	8-22.7	7.6–17.9	8-17.9
HAQL							
Age group 8–11 years			13	10			
Age group 12–16 years			4	7			
Delayed diagnosis	9		4		8	1	
Type of lesion							
Perineal fistula	43		17		26		
“Low malformation”**	1				1		
Associated malformations							
Esophageal atresia							
Cardiac malformations	5		2		3	2	
Renal malformations	3					3	
Lumbar vertebrae malformations	2		1		2		
Limb malformations	2 1		1 1		2 1		

*16 of these patients were also included in the BFS

**According to patient record data

### Outcome from the HAQL

**Fig. 2:**
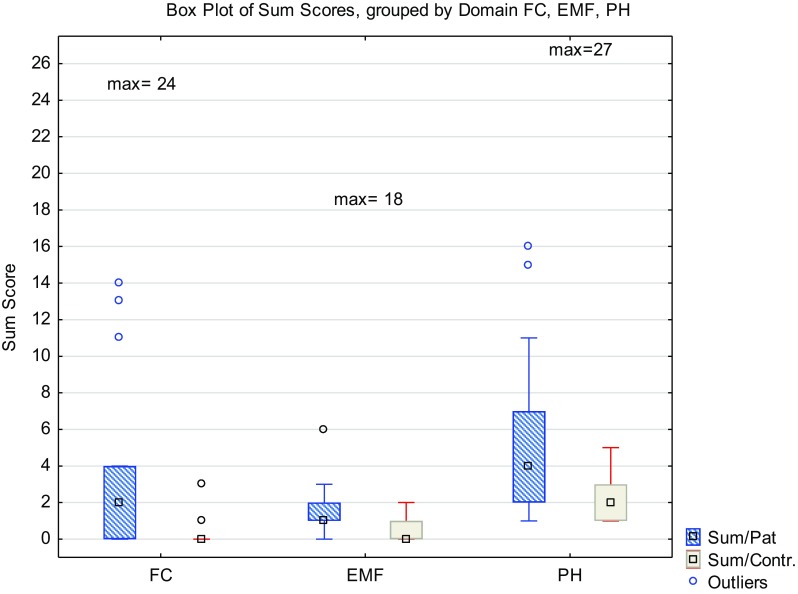
Comparison of sum scores between children with low ARM (pat) and health controls (contr) by domain FC fecal continence, EMF emotional functioning and PH physical function

Compared to the healthy control group, the children and adolescents with low ARM reported significantly lower function in the physical symptom (PH) (*p* = 0.045), fecal continence (FC) (*p* < 0.01) and laxative diet (LD) (*p* < 0.01) (Fig. 2) domains. Differences were also found in the emotional functioning (EMF) domain, in which children with ARM scored lower functioning, although the result was not significant. No systematic differences could be statistically demonstrated in the other domains.

Children and adolescents with low ARM and their mothers were matched on a pair level. The children scored significantly lower function and more symptoms than their mothers in the emotional functioning (EMF) (*p* < 0.01) and physical symptom (PH) (*p* = 0.018) domains. No systematic differences were found between the scoring of the mothers’ and children’s in the other domains.

Both the mothers and fathers were asked to complete the questionnaire in the control group as well as in the index group. Compared to the mothers’ response rate, the response rate among the fathers, particularly in the control group (six fathers), was low. As the answers among the participating fathers were similar to the mothers’ answers, the fathers were not included in the analysis. The domains concerning sexual function (SEF) and constipation (CON) were not analyzed.

In the FC, EMF and UC domains, children from the younger age group (index group 8–11 years) reported more symptoms than those of the older patients (index group 12–16 years), but the differences were not significant.

### Outcome from the BFS

27 families (63%) completed the BFS (Table 1). The overall mean score was 17.2(1.9), which is comparable to the Finnish population with low ARM [18]. 17 of the patients (63%) scored ≥ 17 which is within normal range, and 6 of these, (22%) scored 20. Nine of the patients (33%) scored between 12 and 16 which is a moderate outcome, and one scored 10. The 15 boys scored a slightly higher mean 17.7 (2.8) than the 12 girls, who had a mean of 16.3 (2.0). The younger patients (8–11 years) scored lower with a mean of 16.3 (1.9), compared to the older patients [17.0 (1.6)]. None of these differences were significant.

### Evaluation of the HAQL

Due to the low number of participants (17) in the index group, we were not able to verify or question the different domains in the questionnaire. However, a parsimonious evaluation of the HAQL domains FC, EMF and PH using a Mokken scalability analysis and Rasch analysis showed no obvious conflicts in any of the investigated domains. It should be noted that in most of the matched pair reports, both children and mothers reported “never” (0) when answering and a few mothers reported “I don’t know”.

### Comparison between BFS and HAQL

16 pairs were used in the comparison. No noticeable correlation was found between the BFS and the HAQL domains FC and PH.

### Drop-out analysis

Using descriptive statistics, all participating patients with ARM were compared to non-responding patients using hospital records (Table 1). The groups were similar regarding the patient’s bowel function when comparing the non-participants with ARM and participants with ARM at the most recent check-up.

## Discussion

The results of this present study indicate an impaired QoL among children and adolescents with ARM in some of the HAQL domains. This confirms the findings of previous studies in which adult patients with ARM reported more physical, psychosocial and overall QoL problems compared to control groups [23] and patients with ARM appear to have more problems with soiling, constipation and gas incontinence as well as lower reported QoL compared to control groups [7].

Overall, children and adolescents with low ARM appear to have more physical problems than their healthy peers. They scored significantly lower results in three of the domains of the HAQL questionnaire: physical symptom, fecal incontinence and laxative diet. Differences were also found between the two groups in the emotional functioning domain where patients with low ARM scored worse function, although the results were not significant. Differences in body image and emotional functioning were found to be the most important factors affecting the patients’ QoL [24].

Patients with low ARM are considered to have a good outcome regarding their bowel function. Adult patients with low ARM (perineal fistulas) did not differ much from control group even though many of these patients had a suboptimal function and female patients with perineal fistulas had a worse function compared to male patients with perineal fistulas [25]. Functional impairment is correlated with the severity of the ARM, where patients with a more severe form of the malformation reported impaired bowel function [24, 26]. However, the severity of the malformation did not appear to affect QoL, when patients with cloacal malformation were compared to female patients with milder forms of ARM [27].

The patients in the index group and their mothers scored similar results except for the physical symptoms (PH) and emotional functioning (EMF) domains, where the children reported more symptoms and lower emotional functioning than their mothers. It is well established that pediatric self-reports should be used as standard when measuring HRQoL and other subjective symptoms in children [28] and that self-assessment by children is not synonymous with proxy-reported information [11]. In contrast to our results, proxies tend to underestimate the patients’ QoL and it is common for observers to underestimate the impact of psychological aspects and focus more on physical symptoms [29, 30]. Few studies have been conducted on comparing self- and proxy reports on children with ARM. When generic QoL was measured in children with ARM, Hartman and colleagues found that parents tend to overestimate both physical functioning and emotional functioning [31]; however, when psychosocial aspects among children with high and intermediate ARM were investigated, mothers assessed their children as being sadder and angrier than did the children’s own assessment of themselves [32].

However, the use of proxy informants is relevant in pediatric care, in which patients may be considered unreliable because of their language skills, ability to read, memory or developmental stage [16]. A combination of both self-reports and proxy reports is preferred in QOL assessments [33]. Also, the use of both proxy tools and child self-evaluation tools may be complementary and relevant [14].

The evaluation of the long-term functional outcome in the index group using BFS revealed similar results as found in the Finnish population with low ARM [5, 18]. The Finnish studies revealed differences in bowel function when patients with low ARM were compared to healthy peers. We did not have a comparison group available for the present study, but the similarities between Sweden and Finland and the similar results in the study groups might give us an indication of the bowel function using the BFS of Swedish children with low ARM.

No evident correlation was found between the BFS and the HAQL domains FC and PH. Other studies have shown that patients in all age-groups with impaired fecal function also reported impaired QoL [34, 35]. However, less symptoms do not necessarily imply a better QoL. In a few studies, patients with ARM and fecal incontinence problems did not report more QoL problems compared to the control group [8, 36].

Even if no correlation was seen between the domains investigated in the HAQL instrument and the BFS in the present study, there were similar results in the two questionnaires regarding bowel function and age among the children with low ARM. The younger children (aged 8–11) appeared to have more impaired bowel function compared to the older participants (aged 12–16) in both instruments. The BFS also had an additional age group (17–23), that scored even better results than the previous age groups. These results confirm previous studies, in which children with ARM reported more fecal problems compared to adolescents [36, 37]. Even if bowel function problems tend to improve with age, the adolescents appear to have a poorer QoL regarding their psychosocial function [24, 38], although this could not be confirmed in the present study.

### Methodological discussion

The HAQL questionnaire has, previously been translated into Swedish and adjusted for the target population [13]. When translating and culturally adapting a questionnaire, it is essential to evaluate the questionnaire’s characteristics to verify whether it works as intended. The questionnaire is divided into specific domains. However, this study was too small to fully verify these domains. Instead, an investigation of the item set in each domain was performed to establish how the questions correlated and contributed to the domain and see if the questions fit. Furthermore, the number of items within each domain varies from 2 to 9, which is problematic, as dimensions with less than three items are considered more likely to be unstable [39].

Nevertheless, the FC, EMF and PH domains were basically evaluated to verify whether they could conflict with the original intention of the HAQL. We found no obvious conflicts and the questions appeared to function as intended. Due to non-response to some questions, a straightforward comparison of sum scores would affect the analyses. Thus, for the FC, EMF and PH domains, a transformation to a metric measure via a Rasch approach was performed. Furthermore, the Rasch model was able to include incomplete questionnaires as opposed to the Mokken analysis, which may be troublesome aspect in a small study. The questionnaires of the mothers and children were combined into a set of 34 questionnaires to obtain as much information as possible for the validation. This might be regarded as being a rather unusual approach. However, the pairwise dependency child–mother does not affect the estimation procedure although it will magnify the scalability to some extent. However, it does not conceal the very basic purpose of the analysis.

An additional weakness of the study is that the index group does not cover the entire spectrum of the malformation. The Rasch analysis indicates the difficulty of the questionnaire in capturing the target population. In this case, they appeared to be too healthy for the questionnaire. For example, regarding the LD and CD domains, the answers were mostly zero. Thus, to be able to verify a lack of conflict within the domains, a larger population covering the entire spectrum is necessary.

The relationship between the BFS and the HAQL domains FC and PH was evaluated, but no noticeable correlation was found. Data were collected on different occasions, in some cases several years apart. This could of course, have affected the results as the bowel function seems to improve over age. Only 16 pairs were available for the comparison. A larger sample would have been preferable.

There are difficulties with the original structure of the HAQL as several of the domains are considered weak in its original form [12]. This could largely be explained by the very sparse item set: only two items for certain domains. To obtain a valid, and for the children shorter and easier measure, the developers of the French version of the HAQL have proposed a modified structure in which the new version contains fewer dimensions with only two items in order to achieve a more robust structure for the adolescents and adult versions of the HAQL [40]. They also recommended a structure with fewer items and dimensions developed for children and proxies [14].

The main limitation of this study was the sample size. The drop-out analysis revealed no differences between the non-participants and responding patients regarding their bowel function as recorded in the medical charts at most recent follow-up. We therefore believe that the results have not been biased by the drop-out to any substantial degree. This present study concerns the milder forms of ARM and the participation rate might have been different if more severe forms of the malformations had been included. A previous follow-up study on children with intermediate and high ARM performed at our center had a much higher participation rate [41].

## Conclusions

Children and adolescents with low ARM did not differ regarding their QoL, even though they appeared to have impaired bowel function and worse emotional functioning compared to the healthy control group. The mothers rated optimistically; they underestimated their children’s physical symptoms and overestimated their emotional functioning. It is important for health care providers to also ask children themselves about their wellbeing. The evaluated domains in the HAQL appear to work as intended, but the questionnaire needs further development.
